# Using data from a behavioural survey of men who have sex with men (MSM) to estimate the number likely to present for HIV pre-exposure prophylaxis (PrEP) in Ireland, 2017

**DOI:** 10.2807/1560-7917.ES.2017.22.48.17-00768

**Published:** 2017-11-30

**Authors:** Laura Nic Lochlainn, Kate O’Donnell, Caroline Hurley, Fiona Lyons, Derval Igoe

**Affiliations:** 1 Health Service Executive Health Protection Surveillance Centre, Dublin, Ireland; 2 Health Service Executive Sexual Health and Crisis Pregnancy Programme, Dublin, Ireland

**Keywords:** Men who have sex with men, HIV, Pre-Exposure Prophylaxis, PrEP, behavioural data, Ireland

## Abstract

In Ireland, men who have sex with men (MSM) have increased HIV risk. Pre-exposure prophylaxis (PrEP), combined with safe sex practices, can reduce HIV acquisition. We estimated MSM numbers likely to present for PrEP by applying French PrEP criteria to Irish MSM behavioural survey data. We adjusted for survey bias, calculated proportions accessing testing services and those likely to take PrEP. We estimated 1–3% of MSM in Ireland were likely to present for PrEP.

In Ireland, men who have sex with men (MSM) are at increased risk of sexually acquired HIV infection [1]. A priority action in Europe is to reduce new HIV infections among MSM by improving HIV combination prevention programmes, potentially in part through provision of pre-exposure prophylaxis (PrEP) [2,3]. Across Europe, many countries are working towards implementation of PrEP [4].

Estimating the number likely to present for PrEP is important for informing decision making about PrEP introduction. As recent behavioural data in MSM were available from an online convenience survey, MSM Internet Survey Ireland (MISI) [5], this allowed us to estimate the number of MSM likely to present for PrEP in Ireland.

## Determining PrEP estimates for Ireland

We researched PrEP criteria from Australia [6], England [7], France [8,9], and the United States [10,11], and preliminarily applied those criteria to MISI data to estimate MISI respondents eligible for PrEP. We presented and discussed our estimates and underlying criteria during consultation meetings with clinicians, public health experts and the national PrEP working group, which is a multi-sectoral group, comprised of clinicians, pharmacists, community leaders and public health experts. We obtained consensus that French PrEP criteria were most suited for the purpose of PrEP estimates for Ireland. In this study, we therefore chose variables which were the same as, or the closest fit to French PrEP criteria but restricted our analysis to MISI respondents aged 18–64 years.

PrEP is available in France for men and transgender people over the age of 18 years who have had sex with men and who reported one or more of the following: condomless anal intercourse (CAI) with at least two different sexual partners in the last 6 months; episodes of sexually transmitted infections (STIs) in the past 12 months; multiple post-exposure prophylaxis (PEP) treatments in the last 12 months; or used drugs during sex [8,9].

## Stepwise approach to estimate the number of MSM likely to present for PrEP

**Figure 1:**
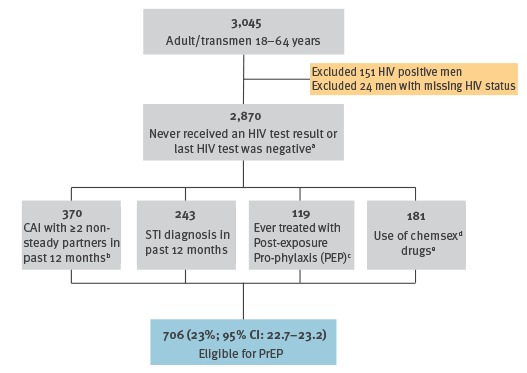
Using data from the MSM Internet Survey Ireland (MISI) and French pre-exposure prophylaxis (PrEP) criteria to estimate the proportion of MISI respondents eligible for HIV PrEP, Ireland, 2017 (n =  3,045 survey respondents)

CAI: condomless anal intercourse; PrEP: pre-exposure prophylaxis; STI: sexually transmissible infection; MISI: Men who have sex with men Internet Survey Ireland (MISI).

**Figure 2:**
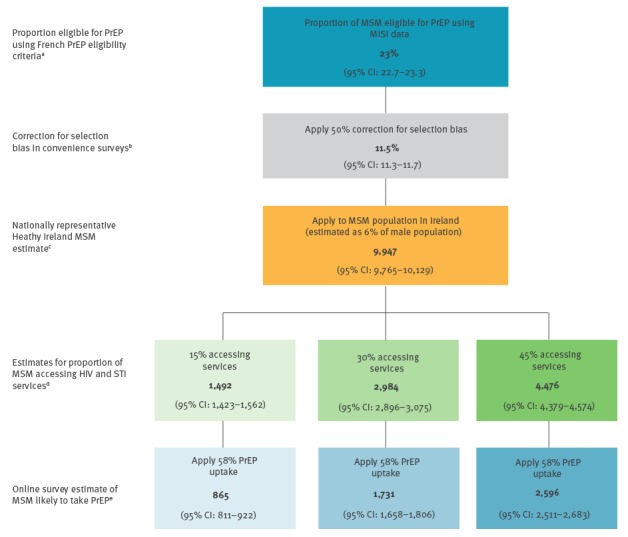
Stepwise approach to estimating the number of men who have sex with men (MSM) who would likely present for HIV pre-exposure prophylaxis (PrEP)^a^ in Ireland, 2017

CI: confidence interval; MSM: men who have sex with men; PrEP: pre-exposure prophylaxis; STI: sexually transmitted infection.

^a^ Estimated proportion of respondents to a behavioural survey, who might present for HIV pre-exposure prophylaxis based on French PrEP eligibility criteria [8,9].

^b^ Based on findings that high-risk MSM were twice as likely to respond to convenience surveys and report more risk behaviours, STI outcomes and HIV testing, compared with probability based surveys [13-15].

^c^ 6% estimate based on [5].

^d^ Applied proportions of MSM accessing STI and HIV testing services in Ireland based on [5,16].

^e^ 58% PrEP uptake rate based on [17].

After estimating the proportion of MISI respondents eligible for PrEP (Figure 1), we developed a stepwise approach to estimate the MSM population in Ireland likely to present for PrEP in the first year of a PrEP programme, should this be introduced (Figure 2). Using the 2015 Healthy Ireland Survey, which found 6% of men in Ireland reporting that their last sex was with a man [5], we applied this estimate to the Irish male population aged 18–64 years from the 2011 census (n = 1,441,603) [12]. This gave us the estimated number of MSM in Ireland to be 86,498.

Criteria for receiving PrEP in France have been previously described [8,9].

^a^ Number of men who reported to be HIV negative or did not know their HIV status.

^b^ This criterion is the closest fit in the MISI survey to the French PrEP eligibility criterion ‘CAI with two or more partners in the past six months’.

^c^ This criterion is the closest fit in the MISI survey to the French PrEP eligibility criterion ‘multiple post-exposure prophylaxis (PEP) in the past 12 months’.

^d^ Crystal methamphetamine, gammahydroxybutrate (GHB) or gamma-butyrolactone (GBL), mephedrone, ketamine.

^e^ This criterion is the closest fit in the MISI survey to the French PrEP eligibility criterion ‘use of drugs during sexual intercourse’.

Previous research found that high-risk MSM were twice as likely to respond to convenience surveys and report more risk behaviours, STI outcomes and HIV testing compared with probability based surveys [13-15]. Therefore, subsequent to estimating the proportion of MSM eligible for PrEP using MISI data, we applied a 50% correction factor to our estimate (Figure 2).

The proportion of MSM accessing STI and HIV testing services in Ireland is not available. However, based on previous study findings [5,16], we applied a range of estimates (15%, 30%, 45%) for the proportion of MSM accessing services.

We applied a rate of 58% for the proportion of MSM likely to take PrEP based on results from an online survey of PrEP awareness and acceptability among MSM in Scotland, Wales, Northern Ireland and the Republic of Ireland [17]. This survey targeted HIV-negative/status unknown MSM who reported CAI with two or more men in the last year, whereby respondents had similar characteristics to the MISI respondents considered in the PrEP eligible group. 

## Results of PrEP estimates

Applying French PrEP criteria to MISI data, we estimated that 23% (95% confidence interval (CI): 22.7–23.3) of the MISI respondents (n = 3,045) would be eligible to receive PrEP (Figure 1).

In order to adjust for over-reporting, we applied a 50% correction factor to the proportion of MISI respondents eligible for PrEP. Applying the adjusted estimate of 11.5% to the MSM population in Ireland, we estimated that 9,947 MSM (95% CI: 9,765–10,129) in Ireland would be eligible for PrEP (Figure 2).

The application of estimate ranges (15%, 30% and 45%) to account for the proportion of MSM accessing HIV and STI services, further adjusted the estimated number of MSM likely to present for or be offered PrEP to between 1,492 and 4,476 (95% CI: 1,423–4,574) (Figure 2).

Applying 58% for the proportion of MSM likely to take PrEP if requested or offered while accessing HIV and STI services, we estimated that 865–2,596 (95% CI: 811–2,683) MSM would likely present and take PrEP (Figure 2).

This estimate of 865–2,596 MSM (95% CI: 811–2,683) likely to present and take PrEP equates to 1–3% of the MSM population in Ireland aged 18–64 years.

## Discussion and conclusion

Through consultation with experts and community leaders, we were able to establish suitable criteria, data sources and a stepwise approach for estimation of the likely number of MSM to present for PrEP in Ireland. We estimated that 1–3% of the MSM population accessing services in Ireland aged 18–64 years would be likely to present and take PrEP. These estimates are currently being used to inform the pharmacoeconomic evaluation of Truvada for PrEP in line with the reimbursement process for medicines in Ireland.

Our findings are subject to some limitations. These estimates are limited to men aged 18–64 years due to the age distribution of MISI respondents. However, if made available, PrEP would not have an upper age limit for eligibility. Also, these estimates are based on the proportion of MSM accessing services. However, if MSM who are not currently accessing services come forward for PrEP, this will increase the number presenting for PrEP. We were unable to apply the exact French PrEP criteria to some MISI variables, which might have under- or over-estimated our findings. The estimate for the proportion of MSM likely to take PrEP is based on findings from an online survey [17] which may not reflect actual uptake when an individual is presented with the option of taking PrEP. Although the MISI survey was large (3,090 respondents), and corrected for over-reporting, responses may still not be representative of the MSM population in Ireland. Finally, the proportion of males in Ireland who are MSM is based on a national probability based survey [5], which may be an over- or under-estimate of the proportion of MSM in Ireland.

These estimates should be reviewed one year post-implementation of PrEP to calculate future projections. It is also important to monitor PrEP uptake to assess its utilisation and to support the development of targeted implementation programmes and policies to increase access for populations most at risk of HIV acquisition.

Given the priority actions within Europe to reduce new HIV infections and improve HIV combination prevention programmes for MSM, other countries may consider replicating the approach we took to estimate the number likely to present for PrEP.

## References

[1] Health Service Executive (HSE). Health Protection Surveillance Centre, HIV in Ireland, 2016*.* Dublin: Health Protection Surveillance Centre; 201*7*.

[2] European Centre for Disease Prevention and Control (ECDC). HIV and STI prevention among men who have sex with men. Stockholm: ECDC; 2015.

[3] World Health Organization (WHO). Guidelines: prevention and treatment of HIV and other sexually transmitted infections among men who have sex with men and transgender people: recommendations for a public health approach 2011. Geneva: WHO; 2011. Available from: http://www.who.int/hiv/pub/guidelines/msm_guidelines2011/en/ 26158187

[4] Coleman R Prins M Options for affordable pre-exposure prophylaxis (PrEP) in national HIV prevention programmes in Europe. Euro Surveill. 2017;22(42):17-00698. 10.2807/1560-7917.ES.2017.22.42.17-00698 29067904 PMC5710114

[5] O’Donnell K, Fitzgerald M, Barrett P, Quinlan M, Igoe D. MISI 2015: Findings from the men who have sex with men internet survey. 2016; Available from: http://www.hpsc.ie/A-Z/SpecificPopulations/MenwhohavesexwithmenMSM/MISI2015/

[6] New South Wales Ministry of Health. Pre-Exposure Prophylaxis of HIV with Antiretroviral Medications. 2016; Available from: http://www1.health.nsw.gov.au/pds/ActivePDSDocuments/GL2016_011.pdf

[7] Field N. Using national surveillance data to estimate numbers eligible for PrEP. Presentation at European Centre for Disease Prevention and Control PrEP meeting; April 2016.

[8] Association AIDES. What is PrEP [La Prep c'est quoi?]. AIDES; 2016; Available from: http://www.aides.org/info-sante/prep#prep

[9] Molina J. PrEP Implementation in France: Challenges, Opportunities and Lessons Learnt. Presentation at European Centre for Disease Prevention and Control PrEP meeting; April 2016.

[10] US Public Health Service. Preexposure prophylaxis for the prevention of HIV infection in the United States - 2014: a clinical practice guideline. In Washington, DC: US Public Health Service; 2014.

[11] Smith DK Van Handel M Wolitski RJ Stryker JE Hall HI Prejean J Vital Signs: Estimated Percentages and Numbers of Adults with Indications for Preexposure Prophylaxis to Prevent HIV Acquisition--United States, 2015. MMWR Morb Mortal Wkly Rep. 2015;64(46):1291-5. 10.15585/mmwr.mm6446a4 26606148

[12] Central Statistics Office (Ireland). Census 2011: StatBank table CD202: Population by Age Last Birthday, At Each Year of Age, Sex and Census Year. 2011. Available from: http://www.cso.ie/px/pxeirestat/Statire/SelectVarVal/Define.asp?maintable=CD202&PLanguage=0

[13] Prah P Hickson F Bonell C McDaid LM Johnson AM Wayal S Men who have sex with men in Great Britain: comparing methods and estimates from probability and convenience sample surveys. Sex Transm Infect. 2016;92(6):455-63. 10.1136/sextrans-2015-052389 26965869 PMC5013102

[14] Evans AR Wiggins RD Mercer CH Bolding GJ Elford J Ross MW Men who have sex with men in Great Britain: comparison of a self-selected internet sample with a national probability sample. Sex Transm Infect. 2007;83(3):200-5. 10.1136/sti.2006.023283 17135330 PMC2659092

[15] Marcus U Hickson F Weatherburn P Schmidt AJ EMIS Network Prevalence of HIV among MSM in Europe: comparison of self-reported diagnoses from a large scale internet survey and existing national estimates. BMC Public Health. 2012;12(1):978. 10.1186/1471-2458-12-978 23151263 PMC3526585

[16] McBride O, Morgan K, McGee H. Irish Contraception and Crisis Pregnancy Study 2010 (ICCP-2010). A Survey of the General Population. Crisis Pregnancy Programme Report No. 24. Dublin: Health Service Executive Crisis Pregnancy Programme (CPP), 2012.

[17] Frankis JS Young I Lorimer K Davis M Flowers P Towards preparedness for PrEP: PrEP awareness and acceptability among MSM at high risk of HIV transmission who use sociosexual media in four Celtic nations: Scotland, Wales, Northern Ireland and The Republic of Ireland: an online survey. Sex Transm Infect. 2016;92(4):279-85. 10.1136/sextrans-2015-052101 26801225

